# Draft Genome Sequences of Lactobacillus salivarius A3iob and Lactobacillus johnsonii CRL1647, Novel Potential Probiotic Strains for Honeybees (Apis mellifera L.)

**DOI:** 10.1128/MRA.00975-18

**Published:** 2018-08-16

**Authors:** Marcela Carina Audisio, Leonardo Albarracín, Maria Julia Torres, Lucila Saavedra, Elvira Maria Hebert, Julio Villena

**Affiliations:** a Instituto de Investigaciones para la Industria Química (INIQUI), Consejo Nacional de Investigaciones Científicas y Técnicas (CONICET), Universidad Nacional de Salta, Salta, Argentina; b Centro de Referencia para Lactobacilos (CERELA), Consejo Nacional de Investigaciones Científicas y Técnicas (CONICET), San Miguel de Tucumán, Argentina; c Laboratorio de Computación Científica, Facultad de Ciencias Exactas y Tecnología, Universidad Nacional de Tucumán, San Miguel de Tucumán, Argentina; Georgia Institute of Technology

## Abstract

This report describes the draft genome sequences of Lactobacillus salivarius A3iob and Lactobacillus johnsonii CRL1647, probiotic strains isolated from the gut of honeybee Apis mellifera workers. The reads were generated by a whole-genome sequencing (WGS) strategy on an Illumina MiSeq sequencer and were assembled into contigs with total sizes of 2,054,490 and 2,137,413 bp for the A3iob and CRL1647 strains, respectively. The draft genome sequences of L. salivarius A3iob and L. johnsonii CRL1647 will be useful for further studies of the specific genetic features of these strains and for understanding the mechanisms of their probiotic properties.

## Abstract (precis)

This report describes the draft genome sequences of Lactobacillus salivarius A3iob and Lactobacillus johnsonii CRL1647, probiotic strains isolated from the gut of honeybee Apis mellifera workers. The reads were generated by a whole-genome sequencing (WGS) strategy on an Illumina MiSeq sequencer and were assembled into contigs with total sizes of 2,054,490 and 2,137,413 bp for the A3iob and CRL1647 strains, respectively.

## ANNOUNCEMENT

Lactobacillus salivarius subsp. salivarius A3iob and Lactobacillus johnsonii CRL1647 were isolated from the gut of honeybee Apis mellifera workers and selected among several isolates due to their antimicrobial properties and high lactic acid production (1, 2). Both Lactobacillus strains elicited probiotic properties when administered individually to commercial productive hives of the honeybee Apis mellifera L. Lactobacillus strains generated a higher number of bees through stimulation of the queen's egg laying (1–3). An increase in the resistance to parasitic mite (Varroa spp.) and microsporidian parasite (Nosema spp.) infections has been also observed after the administration of A3iob or CRL1647 strains (1, 4). These beneficial properties have been evaluated and confirmed in different ecoregions of Argentina and under several different beekeeping practices (5).

L. salivarius A3iob and L. johnsonii CRL1647 were cultured for 12 h at 37°C (final log phase) in de Man-Rogosa-Sharpe broth (MRS; Oxoid, Cambridge, UK), and genomic DNA isolation was performed as described by Azcárate-Peril and Raya (6). Draft genome sequences of both bacteria were obtained with an Illumina MiSeq platform using the 2 × 150-bp paired-end read length sequencing protocol. The L salivarius A3iob and L. johnsonii CRL1647 data sets contain 2,216,287 and 2,391,328 reads, respectively. The raw sequence data were analyzed by FastQ for quality control purposes. These reads were *de novo* assembled with SPAdes version 3.11.1 (7). The A3iob strain contained 12 contigs (2,054,490 bp, 34.6% G+C content, 114.0× coverage), while the CRL1647 strain contained 38 contigs (2,137,413 bp, 34.4% G+C content, 72.0× coverage). The Rapid Annotations using Subsystems Technology (RAST) server and standalone Prokka (rapid prokaryotic genome annotation) program were used for functional annotation of predicted genes (8, 9). A total of 1,911 coding sequences, 61 tRNAs, 20 rRNAs, 3 noncoding RNAs (ncRNAs), and 1 CRISPR array were annotated in the L salivarius A3iob genome. In the L. johnsonii CRL1647 genome, 1,762 coding sequences, 30 tRNAs, 6 rRNAs, 3 ncRNAs, and 2 CRISPR arrays were found.

The genomes were further analyzed with BAGEL4 for the detection of bacteriocin genes (10). Salivaricin and enterolysin A genes were found in the A3iob genome, while helveticin J, thermophilin A, and enterolysin A genes are present in the CRL1647 genome. Both Lactobacillus strains contain genes encoding fibronectin binding protein that could be involved in their probiotic effect. In addition, clusters of genes involved in the biosynthesis of pyridoxine, folate, biotin, and riboflavin were found in the A3iob genome, while the CRL1647 genome harbors genes involved in folate, thiamine, and riboflavin biosynthesis.

The draft genome sequences of the A3iob and CRL1647 strains will be useful for further studies of their specific genetic features and for understanding the mechanisms of their probiotic properties.

### Data availability.

The draft genome sequences of Lactobacillus salivarius subsp. salivarius A3iob and Lactobacillus johnsonii CRL1647 have been deposited as whole-genome shotgun sequencing projects at DDBJ/EMBL/GenBank under the accession numbers QFAS00000000 and QFBA00000000, respectively. The versions described in this paper are the first versions, QFAS01000000 and QFBA01000000.
